# TNF Receptor 2 Makes Tumor Necrosis Factor a Friend of Tumors

**DOI:** 10.3389/fimmu.2018.01170

**Published:** 2018-05-28

**Authors:** Yuqiao Sheng, Feng Li, Zhihai Qin

**Affiliations:** ^1^Medical Research Center, The First Affiliated Hospital of Zhengzhou University, Zhengzhou, China; ^2^Biotherapy Center, The First Affiliated Hospital of Zhengzhou University, Zhengzhou, China

**Keywords:** tumor necrosis factor, TNF receptor 2, tumor, myeloid-derived suppressor cells, regulatory T cells, macrophages

## Abstract

Tumor necrosis factor (TNF) is widely accepted as a tumor-suppressive cytokine *via* its ubiquitous receptor TNF receptor 1 (TNFR1). The other receptor, TNFR2, is not only expressed on some tumor cells but also on suppressive immune cells, including regulatory T cells and myeloid-derived suppressor cells. In contrast to TNFR1, TNFR2 diverts the tumor-inhibiting TNF into a tumor-advocating factor. TNFR2 directly promotes the proliferation of some kinds of tumor cells. Also activating immunosuppressive cells, it supports immune escape and tumor development. Hence, TNFR2 may represent a potential target of cancer therapy. Here, we focus on expression and role of TNFR2 in the tumor microenvironment. We summarize the recent progress in understanding how TNFR2-dependent mechanisms promote carcinogenesis and tumor growth and discuss the potential value of TNFR2 in cancer treatment.

## Introduction

The 34 kDa pleiotropic cytokine tumor necrosis factor (TNF) is a type II transmembrane protein important in carcinogenesis, cancer progression, and metastasis, as well as in immunity (1–3). Connecting a wide variety of cell types, TNF constitutes itself a central player in the multi-faceted tumor microenvironment. TNF can exert both tumor-promoting and -suppressing roles, and those distinct effects are transmitted by two receptors, TNF receptor 1 (TNFR1) and TNFR2 (4–8). Although the role of TNFR2 is less well understood, many reports indicated it as crucial in tumors (Table 1). This review does not aim to give all details of TNFR2-mediated cellular and molecular mechanisms. We specifically emphasize how tumor progression is accelerated after TNFR2 activation on tumor and immune cells within a tumor and briefly discuss the outcome of treatments targeting TNFR2.

**Table 1:** Tumor development promoted by TNFR2-mediated signaling in tumor or tumor-associated cells.

Cancer type	Impacts of TNF receptor 2 (TNFR2) expression on various cells
Breast cancer	Promoting tumor cell growth (49); inhibiting programmed death of tumor cells (48); stabilizing myeloid-derived suppressor cells (MDSC) (91); and relating to suppressive function of regulatory T (T_reg_) cells (82)
Colon cancer	Advancing carcinogenesis of epithelia cells (59–61); promoting tumor cell proliferation (55, 56); enhancing angiogenesis by upregulating VEGF-A in tumor cells (56); inducing cancer-associated fibroblasts (100); activating T_reg_ cells (78, 105); and supporting metastasis (92)
Cervical cancer	Facilitating tumorigenesis (58)
Fibrosarcoma	Promoting MDSC accumulation (85)
Liver cancer	Expanding T_reg_ cells (78) and promoting tumoral accumulation of MDSC by inducing specific chemokine receptor (87)
Lung cancer	Helping to form metastasis niche by stabilizing MDSC (92); promoting VEGF release and anti-apoptotic ability of tumor cells (50, 51); and associating with inhibitory effects of T_reg_ cells (67, 82)
Melanoma	Maintaining T_reg_ cells (71); contributing to T cell exhaustion (80)
Ovarian cancer	Acting as oncogene in tumor cells (104); expanding T_reg_ cells (104); and promoting T_reg_ cells to impair T-helper 1 immunity (77)
Renal cancer	Driving proliferation of tumor stem cells (54) and accelerating tumor cell division (53, 79)
Skin cancer	Advancing malignant transformation of epidermal cells (40)
Plasmacytoma	Driving MDSC expansion (85)
Lymphoma	Enhancing angiogenesis by inducing interleukin-6 secretion from malignant cells (52) and augmenting activation-induced death of cytotoxic T cells (79)
Leukemia	Relating to T_reg_ cell expansion (102, 103)

*References exploring biological functions of TNFR2 in tumorigenesis, tumor progression, anti-apoptosis, or non-specified other processes in human cancer and murine tumor models within the last 10 years*.

## TNF Receptor 1 and TNF Receptor 2

TNF receptor 1 (p55 or CD120a) and TNFR2 (p75 or CD120b) are type I transmembrane receptors. TNFR1 shows extensive expression, whereas TNFR2 expression is limited to immune cells and a few other cell types (7, 9, 10). TNFR1 and TNFR2 have similar extracellular TNF-binding structures characterized by four repeated cysteine-rich domains (CRDs) (CRD1 also called pre-ligand binding assembly domain, CRD2, CRD3, and CRD4) but have different intracellular domains (11, 12). Most critical for the diverse biological effects of the two receptor subtypes is the lack of the intracellular death domain in TNFR2. Hence, TNF promotes apoptosis *via* binding to TNFR1 but exerts pro-survival effects *via* TNFR2 (4, 5, 13). After being engaged by extracellular TNF, TNFR1 recruits and clusters the adaptor protein TNFR1-associated death domain protein (TRADD) and the downstream caspases (14–16). This finally leads to programmed cell death. In contrast, activated TNFR2 results in recruitment of the TNF receptor-associated factor (TRAF) 2 and stimulates the pro-survival nuclear factor (NF)-κB pathway (17). TNFR2 has a high affinity to membrane-bound TNF and can deliver TNF to TNFR1 (18–21). Only by this cooperation, TNFR2 can feed a cell to its death (22).

## TNF Receptors and the Common Nuclear Factor-κB (NF-κB) Pathway

Nuclear factor-κB is activated by both TNF receptor subtypes. Upon stimulation by its ligands including TNFα or lymphotoxin, TNFR1 forms a complex with the adaptor TRADD at the plasma membrane (23, 24). TRAF2 is transported and clustered into the complex that recruits the cellular inhibitor of apoptosis 1 and 2 (cIAP1/2) proteins (25–27). Together with TRAF2, cIAP1/2 proteins degrade the TRADD-bound ubiquitinated receptor interacting protein (RIP) 1. Multiple ubiquitination of RIP1 and the NF-κB essential modulator [NEMO; also called IκB kinase (IKK)γ] engages the kinase TAK1 to the NEMO-containing IKK complex (5). IKKβ in the IKK complex becomes phosphorylated and phosphorylates the NF-κB inhibitor IκBα that is subsequently cleaved. Released NF-κB translocates into the nucleus and induces target gene expression.

The detailed mechanisms of how TNFR2 induces NF-κB remain more elusive. So far, only TRAF2 is clear as the key component. Different from TNFR1, TNFR2 directly interacts with TRAF2 (28). Activated TNFR2 binds to TNFR2 through two conserved intracellular domains, the TRAF2-binding motif SKEE (amino acid residues 402–405) and the C-terminal motif (amino acid residues 425–439) (29, 30). TRAF1 and TRAF3 also associate with TNFR2 directly or *via* TRAF2 at the two conserved domains (31–33). Genetic manipulation confirmed those two domains as most critical for TNFR2-induced NF-κB activation.

As mentioned above, TNFR1 and TNFR2 have distinct impacts on individual cell fates although they both regulate the outcomes through NF-κB. It is reported that the receptor crosstalk depends on the relative expression of each receptor. At high expression of TNFR1, low amounts of TNFR2 enhance TNFR1-induced NF-κB activation (34). In contrast, TNFR2 at high levels effectively competes for TRAF2. Consequently, recruitment to the classical TNFR1 pathway and downstream NF-κB activation is compromised. Concentration and conformation of the ligand TNF are also related with the balance between TNFR1 and TNFR2 signals (20, 35). Interestingly, a crosstalk of TNFR1 with TNFR2 strongly affects the cell fate decision. When both TNF receptor isotypes are co-expressed, specific activation of TNFR1 leads to continuous expression of anti-apoptotic factors and barely induced apoptotic pathways. Here, cell death is due to the loss of anti-apoptotic factor expression after TNFR2-dependent TRAF2 degradation and abrogated recruitment of cIAP1/2 to TNFR1 (36–38). If both receptors are activated at same time, the balanced signal transduction of TNFR1 and TNFR2 leads to cell survival (39, 40). The TNFR1–TNFR2 crosstalk is context- and time-dependent, and their intricacy clearly needs further exploration.

## TNFR2 Promoting Tumorigenesis and Progression

**Figure 1:**
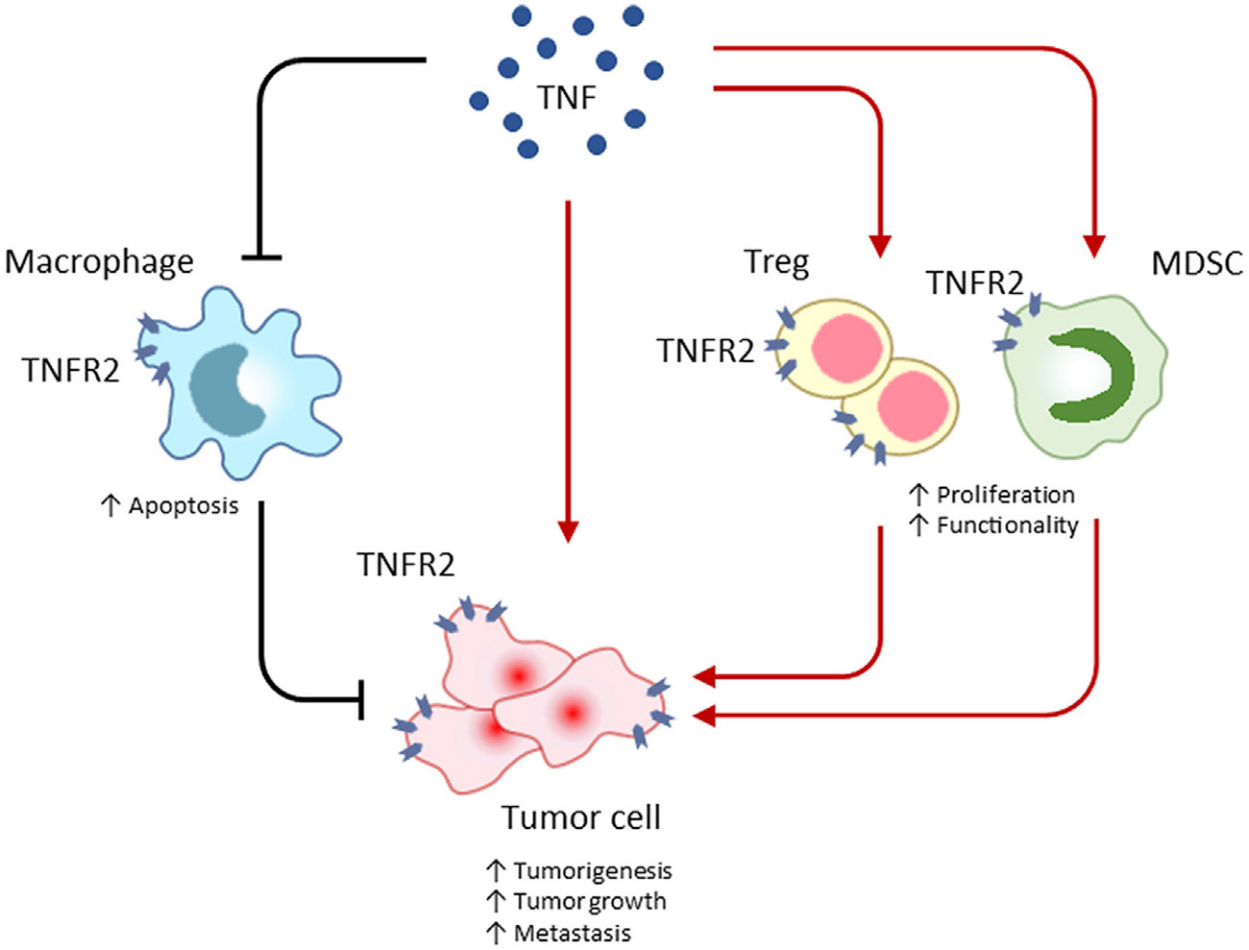
TNF receptor 2 (TNFR2) promotes tumor progression by maintaining a tumor-favoring immune-microenvironment or by facilitating malignant cell proliferation and survival. In the tumor microenvironment, TNFR2 is extensively expressed on many types of cells, including immune cells and malignant cells. TNFR2 often accelerates the malignant transformation and growth of tumor cells, instead of inducing cell death by apoptosis. Similar to tumor cells, TNFR2 protects immunosuppressive regulatory T (T_reg_) cells and myeloid-derived suppressor cells (MDSC) from the death-inducing TNF and consequently enhances proliferation and function of those tumor-promoting cells. Even worse, TNFR2 deteriorates the programmed death of phagocytic macrophages responsible for clearing of tumor cells. Mediating those direct and indirect effects, TNFR2 exacerbates cancer progression.

TNF receptor 2 is implicated in the occurrence and growth of tumors, therapeutic responses, and patients’ prognosis (41–43). In direct and indirect manners, TNFR2 plays important roles in multiple aspects of tumor progression, including the proliferation of tumor cells, the evasion of immune surveillance, the activation of endothelia cells and angiogenesis, and the formation of a pre-metastasis milieu (Figure 1).

### TNFR2 on Tumor Cells and Non-Immune Cells in the Tumor Microenvironment

**Figure 2:**
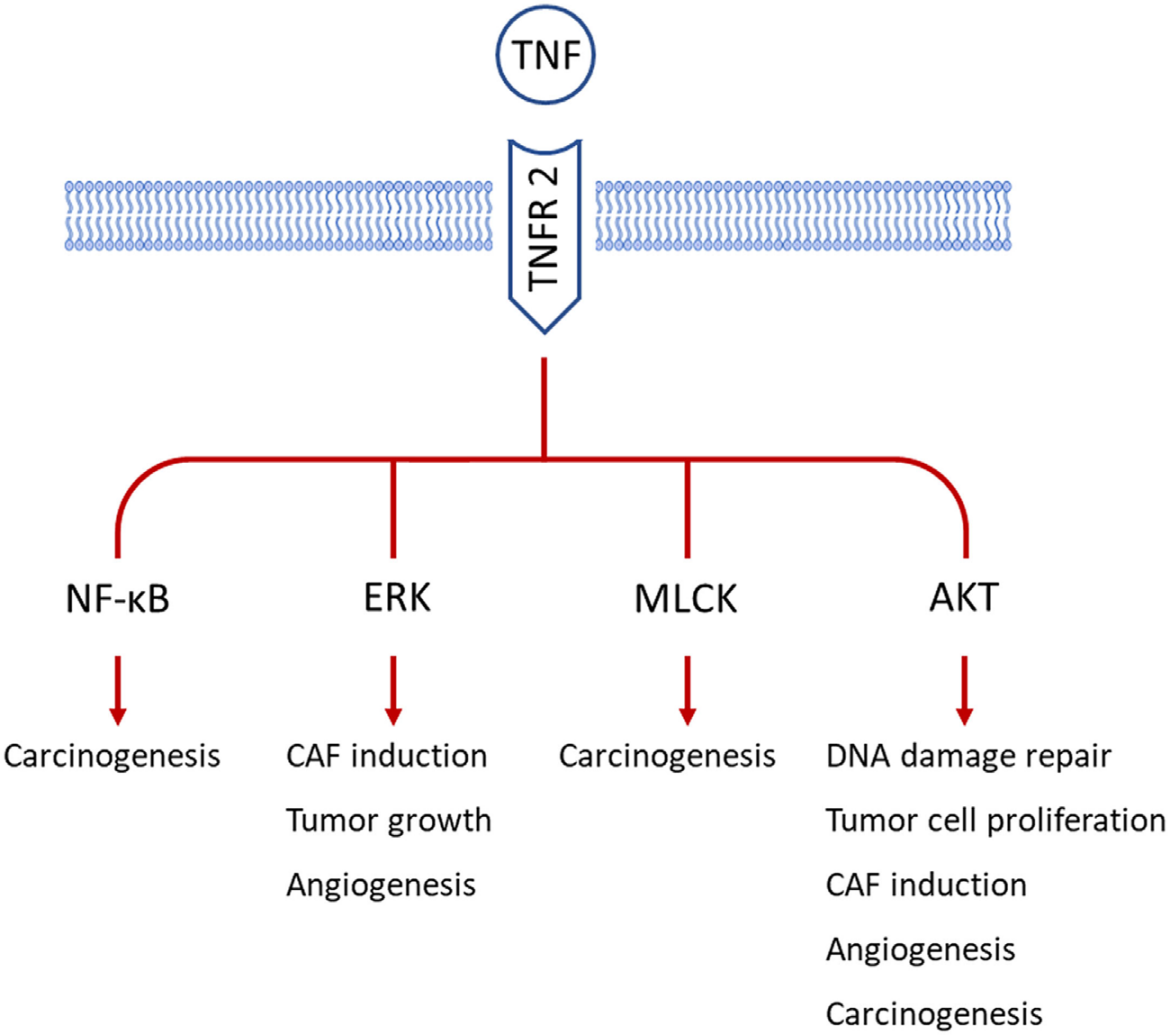
TNF receptor 2 (TNFR2) participates in various processes of tumor development by employing different signal pathways in tumor cells. So far, TNFR2 is reported to be expressed on tumor cells from breast cancer, cervical cancer, colon cancer, and renal cancer. AKT signaling is the major mediator of TNFR2 in carcinogenesis, tumor growth, angiogenesis, and cancer-associated fibroblast (CAF) induction. Besides, myosin light-chain kinase (MLCK) and nuclear factor-κB (NF-κB) are involved in TNFR2-related malignant transformation of epithelial cells. Extracellular signal-regulated kinase (ERK) is also important for the above-mentioned functions of TNFR2.

Several studies have indicated that TNFR2 expression in tumor tissues relates to advanced disease progression and poor clinical outcomes (44–46). TNFR2 is aberrantly expressed on several types of tumor cells (47) and induces tumor progression through several signal transduction cascades (Figure 2). In breast cancer, TNFR2 protects malignant cells from DNA damage *via* the AKT signaling pathway (48) and induces NF-κB *via* p42/p44 mitogen-activated protein kinase (MAPK) to accelerate tumor cell growth (49). Interestingly, blocking TNFR2 is sufficient to diminish TNF-evoked cell growth (49), indicating TNFR2 as more important for tumor progression than the activation of signal kinases including p42/p44 MAPK, JNK, and AKT *via* the ubiquitous TNFR1. Underlining this, loss of TNFR2 results in a large increase of TNF-associated tumor cell death and a significant halt of tumor growth in lung cancer (50).

TNF receptor 2 deficiency in a mouse model of lung cancer not only enhances tumor cell apoptosis but also leads to downregulated pro-angiogenic factors, like vascular endothelial, hepatocyte, and placental growth factors from endothelial progenitor cells (51). Pointing to a general role for TNFR2 in tumor development, TNFR2-deficient mice decrease melanoma cell growth in the same way (51). Additionally, TNFR2 signaling indirectly promotes angiogenesis by inducing interleukin (IL)-6 secretion (52). In renal carcinoma, TNFR2 on endothelial and tubular epithelial cells activates the endothelial/epithelial tyrosine kinase and then upregulates vascular endothelial growth factor receptor-2 (53) or directly promotes cell division (54). Consequently, expression of TNFR2, but not of TNFR1, correlates with the grading of malignancy. In colorectal carcinoma, TNFR2 promotes tumor cell proliferation through the PI3K-AKT pathway (55, 56) or *via* NF-κB activation (57). These studies imply that TNFR2 directly enhances tumor growth, but TNFR2 is also involved in malignant transformation (58). In animal models of chronic inflammation, TNFR2 induces NF-κB activation in epithelial cells that subsequently leads to carcinogensis (59–61).

### TNFR2 on T Cells

TNF receptor 2 not only affects tumor cells but also regulates tumor-infiltrating immune cells. The resulting immunosuppressive microenvironment supports tumor development. Regulatory T (T_reg_) cells are the central player in regulating tumor-specific immune responses (62–64). Hence, T_reg_ cells also represent the most important tumor-promoting cell type and are most extensively studied. TNFR2 expressed on T_reg_ cells indicates the maximally suppressive subset (47, 65–68) and relates to poor prognosis of patients (69). TNFR2 mediates the effects of TNF on CD4^+^ forkhead box (Fox)P3^+^ T_reg_ cells (70, 71). TNFR2 promotes the development of T_reg_ cells in thymus (72), the expansion of differentiated T_reg_ cells (73), and mediates the activation effects of TNF on T_reg_ cells (70, 71). It leads to activation, expansion, and phenotypic stability of the strongly suppressive T cells (74), partially through an epigenetic mechanism that demethylates the *Foxp3* gene (75). TNFR2 is highly expressed on resting and activated T_reg_ cells compared to their FoxP3^−^ counterparts (65). TNF expands the TNFR2^+^ T_reg_ subset and augments the IL-2-induced induction of signal transducer and activator of transcription-5 to increase the suppressive function. Consistently, TNFR2^+^ T_reg_ cells comprise the most highly suppressive subset of T_reg_ cells (67). T_reg_ cells within the tumor microenvironment show higher expression of TNFR2 than T_reg_ cells from normal tissues or the periphery (76).

Emphasizing the clinical relevance of those findings in mouse models, T_reg_ cells infiltrating human tumors have high levels of TNFR2 expression and maximal suppressive capacity (77). Increased TNFR2 in T_reg_ cells enhances TNF-dependent T_reg_-cell proliferation and suppressive effects in tumors susceptible to anti-TNF treatment (78). In lung cancer patients, expression of TNFR2 strongly correlates with the transcription factor FoxP3 than the expression of CD25 (69). TNFR2 expression levels on T_reg_ cells closely associate with lymphatic invasion, distant metastasis, and advanced clinical stage (69). This not only underlines the functional importance of effective T_reg_ cell-mediated control of tumor-specific immune responses but also suggests TNFR2 as a more appropriate marker for tumor-resident T_reg_ cells compared to integrin-αE (CD103) (67).

Taken together, the abundance and strong immunosuppressive capacity point to TNFR2^+^ T_reg_ cells as critical in promoting tumor progression and metastasis.

Although TNFR2 is preferentially expressed on T_reg_ cells, the expression of TNFR2 can be induced or up-regulated on CD8^+^ T cells and conventional CD4^+^ FoxP3^−^ (T_con_) T cells.

In CD8^+^ T cells, TNFR2 can elicit activation-induced cell death (79). It also may upregulate the expression of the inhibitory receptor Tim3 (80). Both direct mechanisms further hamper the efficacy of cytotoxic T cells.

Activation of TNFR2 on T_con_ cells can lead to enhanced tumoricidal effects (81). TNFR2 on T_con_ cells also activates those effector T cells making them resistant to T cell-mediated suppression (82). However, T_reg_ cells within the tumor microenvironment demonstrate much higher expression levels of TNFR2 that overcome this resistance and maintain the local dominance of immunosuppression (77).

### TNFR2 on Myeloid-Derived Suppressor Cells (MDSC)

Myeloid-derived suppressor cells are a heterogeneous population of immature myeloid cells mainly characterized by their strong immunosuppressive capacity. In healthy individuals, myeloid cells outside the bone marrow are mainly matured into granulocytes or monocytes (83, 84). Lineage-specific differentiation fails in chronic inflammation or malignancy, and this associates with a potent immunosuppressive function in the resulting myeloid cells (21, 85). These cells appear in peripheral tissues and also represent an important subset of cells in the tumor microenvironment that promotes tumor growth (83). TNF signaling is believed to be critical for MDSC to accumulate and perpetuate their immature state (86, 87). Elevated TNF in an inflammatory milieu augments MDSC accumulation and immunosuppression, whereas TNF antagonists reduce the inhibitory function of MDSC and support differentiation into dendritic cells or macrophages (88).

The feature of TNF to promote immunosuppression is intimately related to TNFR2. TNFR1 and TNFR2 double-negative mice spontaneously reject implanted tumors. This correlates with decreased accumulation of MDSC, which is mainly mediated by TNFR2, but not by TNFR1 (85). TNFR2-mediated signaling increases the induction of MDSC from bone marrow cells and inhibits the apoptosis of MDSC through c-FLIP upregulation and caspase-8 inhibition. TNFR2 also directs the suppressive functions of MDSC (89). In monocytic MDSC, TNFR2 deficiency compromises the development of MDSC and reduces the production of immunosuppressive factors, like NO and IL-6 (89). Additionally, TNFR2 is important for the production of the immunosuppressive factors, IL-10 and transforming growth factor-β (90). The p38 MAPK-NF-κB axis is indispensable for the process of TNFR2-transmitted signals in MDSC. Inhibition of this axis in TNFR2^+^ MDSC stimulated with TNF could reverse T-cell suppression (91). The induction of suppression-related markers, including arginase-1, inducible NO synthase, NO, reactive oxygen species, IL-10, and transforming growth factor-β clearly correlates with the activation of the p38MAPK-NF-κB pathway *via* TNFR2 (91). All reports support the assumption that TNFR2 promotes primary tumor growth by maintaining the naïve state and enhancing the suppressive character of MDSC to control tumor-specific T-cell responses.

TNF receptor 2-expressing MDSC also contribute to metastasis. TNFR2 deficiency reduces the liver metastasis of lung cancer (92). In a mouse model, TNFR2^−/−^ MDSC fail to accumulate in pre-metastatic lesions and show reduced expression of the suppressive arginase-1. Of note, the loss of TNFR2 also alleviates T_reg_-cell infiltration into metastasis sites of human lung cancer (92). We conclude that TNFR2 coordinates T_reg_ cells and MDSC in original tumor growth as well as in metastasis.

### TNFR2 on Macrophages

Macrophages are the most dominant innate immune cell type in tumor control, and they are the main sources of TNF (93). Macrophages also simultaneously express TNFR1 and TNFR2, although the effects of TNFR2 on macrophages remain unclear. Similar to the immature MDSC, activation of TNFR2 on macrophages induces the p38 MAPK-NF-κB pathway (94). TNFR2 on tumor-associated macrophages correlates with malignancy grades in human triple-negative breast cancer and is thought to participate in metastasis (95).

Prompting to the significance of TNF receptor crosstalk discussed above, the TNFR2 also takes part in inducing macrophage death by necroptosis upon TNF-induced TNFR1 activation upon contact with pathogen (22). Without TNFR2 signaling, the induced necroptosis is reversed. Although not shown so far for tumors, TNF-related macrophage death may represent an alternative way of how TNFR2 signaling in macrophages might contribute to tumor progression.

## Targeting TNFR2 for Tumor Therapy

TNF receptor 2 is mainly expressed on malignant cells and in the immunosuppressive cell compartment within the tumor microenvironment. It is involved in promoted tumor development and facilitated metastasis. Hence, TNFR2 represents an attractive target for tumor treatment.

Specifically, blocking the ligand TNF is one option. Due to the higher expression of TNFR2 relative to TNFR1 in tumor and tumor-associated cells, TNF is likely to have a tumor-promoting function instead of an inhibitory impact. TNF ablation effectively reduces tumor growth (96).

Of note, activating TNFR2 on tumor-promoting cell types, such as fibroblasts might limit tumor cell invasion and metastasis and improve tumor therapy (97–100).

Depleting TNFR2^+^ T_reg_ cells augmented the efficacy of chemotherapy in preclinical studies (101). In a clinical trial with acute myeloid leukemia patients, patients received the demethylating agent, azacitidine, and the histone deacetylase inhibitor, panobinostat, which effectively eliminated TNFR2^+^ T_reg_ cells in peripheral blood and bone marrow (102). These TNFR2^+^ T_reg_ cells were earlier found as potent suppressive immune cell subset with enhanced migratory ability that promote disease progression and hamper tumor therapy (65, 102). Beneficial clinical responses came from more active effector T cells as determined from increased production of interferon-γ and IL-2. A combination of azacitidine with lenalidomide decreasing TNFR2 expression and activity in T_reg_ cells may improve clinical outcomes in hematological malignancies (103).

More recently, antibodies specifically blocking TNFR2 were developed for tumor therapy. TNFR2 is abundant on tumor cells and tumor-infiltrating T_reg_ cells in ovarian cancer (104). Here, antagonistic antibodies to TNFR2 suppress TNF-induced T_reg_-cell activation and reduce amount as well as immunosuppressive function of T_reg_ cells (104). They inhibit NF-κB activation, hence the T_reg_ cell expansion and immunosuppression but synergistically directly induce tumor-cell death (104). This study showed that targeting TNFR2 on T_reg_ cells was well tolerated. It mostly affected the tumor-infiltrating T_reg_ cells that express much higher levels of TNFR2 than normal T_reg_ cells. A concomitant administration of TNFR2-neutralizing antibody and a toll-like receptor agonist has the potential to further improve the therapeutic effectiveness (105).

## Concluding Remarks

A strongly immunosuppressive microenvironment is a major obstacle in tumor therapy. Over the last decade, immunotherapies using checkpoint blockade and engineered T cells have gained great success. However, many patients fail to benefit from these therapies. One important reason for the ineffectiveness is the focus on evoking cytotoxic T-cell responses that overlooks the impact of the immunosuppressive cell compartment. Therapy-related changes in the tumor environment often enhance immunosuppressive effects and finally result in a failure of therapy. Considering this, we need to emphasize the immunosuppressive cells and factors in tumor treatment. Here, TNF and its diverse effects mediated by TNFR1 or TNFR2 provide a clue.

Tumor necrosis factor is abundant in any tumor microenvironment. This cytokine is usually involved in anti-tumor responses. However, TNFR2 may convert the anti-tumor effect into tumor-promoting function. TNFR2 expression is limited to several cell types that include tumor and immune cells (Figure 1). Tumor cells highly expressing TNFR2 resist TNF-induced cell death *via* binding of the ligand to the TNFR2. TNFR2 is not only highly expressed on tumor cells but also on immunosuppressive cells, including T_reg_ cells and MDSC. Thus, TNFR2 is tightly related with the immunoinhibitory capacities of tumor-promoting cells.

All these specific properties of TNFR2 make it an ideal candidate for targeted tumor therapy. Several studies targeting TNFR2 already proved its great potential in treating tumor. Future investigations will provide more detailed knowledge on all facets and on the cell-type dependency of TNFR2’s immunosuppressive effects that we need to translate it into the treatment of malignant diseases.

## Author Contributions

YS, FL, and ZQ wrote the manuscript; ZQ critically revised the manuscript.

## Conflict of Interest Statement

The authors declare that the research was conducted in the absence of any commercial or financial relationships that could be construed as a potential conflict of interest.
